# Efficient photocatalytic reduction of p-nitrophenol under visible light irradiation based on Ag NPs loaded brown 2D g-C_3_N_4_ / g-C_3_N_4_ QDs nanocomposite

**DOI:** 10.1007/s11356-023-30010-z

**Published:** 2023-10-24

**Authors:** Sandy Maged, Ola M. El-Borady, Hamza El-Hosainy, Maged El-Kemary

**Affiliations:** Nano Sensor Group, Institute of Nanoscience and Nanotechnology, Kafrelsheikh University, Kafr ElSheikh, 33516 Egypt

**Keywords:** g-C_3_N_4_, Quantum dots, Silver nanoparticles, Photocatalytic, p-Nitrophenol, Visible light

## Abstract

Recently, low-cost graphitic carbon nitride (g-C_3_N_4_) revealed high photocatalytic activities and provided solutions to environmental pollution. In this study, we synthesized brown mesoporous 2D g-C_3_N_4_ by calcination dicyandiamide with pluronic P123. This is followed by loading of Ag NPs on the prepared 2D g-C_3_N_4_ by photodeposition process. After that, a ternary composite 2% Ag/ 2D g-C_3_N_4_ / g-C_3_N_4_ QDs heterojunction photocatalyst has been successfully prepared. The prepared nanomaterials were comprehensively characterized by various analysis techniques such as XRD, UV–Vis., BET, XPS, SEM, TEM. This new system exhibited a large surface area with porous structure and a wide absorption of visible light. The results verified that Ag NPs decoration enhanced the charge separation of photo-generated carriers of g-C_3_N_4_ 2D and g-C_3_N_4_ QDs, promote significant enhancement in the photocatalytic activity for reduction of p-nitrophenol with a rate constant (k) value of 0.49729 / min in 6 min. This rate is about two-fold higher than that observed for pure g-C_3_N_4_ 2D and g-C_3_N_4_ QDs as well as shows an improvement compared to 2% Ag/ g-C_3_N_4_ 2D and g-C_3_N_4_ 2D/ g-C_3_N_4_ QDs. The results open the door to design highly efficient 2D/0D nanocomposite photocatalysts for a wide variety of environmental applications.

**Supplementary Information:**

The online version contains supplementary material available at 10.1007/s11356-023-30010-z.

## Introduction

Sustainably securing water supplies is increasingly recognized as a challenge for people, industry, and the environment. The challenge is likely due to environmental and global changes, industrial, domestic, and agricultural activities. Synthetic organic dye pollution is one of the major pollutants in the industrial sector. In this context, nitroaromatic compounds have been considered one of the most harmful environmental organic pollutants (Ju and Parales 2010; Spain 1995). P-nitrophenol (PNP) is one of such nitro-compound, which is widely used in industrial and agricultural processes, particularly in drug, dye, and pesticide manufacturing (Feng et al. 2013; Rocha et al. 2018; Yang et al. 2010) and found in industrial effluents because they are very soluble and stable. PNP is extremely harmful to human health; due to its toxic effects leading to a cancer (Yu et al. 2017). Therefore, numerous strategies have been developed for removing these organic pollutants from industrialized sewage, involving physisorption, chemical precipitation, membrane filtration, photocatalytic degradation, biological remedies, and catalytic decomposition, advanced oxidation processes (AOPs) (Chen et al. 2023; Peng et al. 2023; Tang et al. 2023) and enzymatic degradation (Guo et al. 2023). Heterogeneous photocatalysis is a promising approach that harnesses solar light to effectively oxidize contaminants under reasonable conditions (El-Hosainy et al. 2022; El-Sheshtawy et al. 2020; Ghubish et al. 2022; Shoueir et al. 2019; Dileepkumar et al. 2022; Prabagar et al. 2022).

Graphitic carbon nitride (g-C_3_N_4_) is one of the promising semiconductor photocatalyst materials with a layered structure that has a lot of covalent and hydrogen bonds and functions as an organic conjugated semiconductor photocatalyst. G-C_3_N_4_ has gained great attraction as a semiconductor in various applications due to its high stability, cost-effectiveness, excellent performance for solar energy use and a small band gap (2.82 eV) (Chen et al. 2023; Torad et al. 2021; Wang et al. 2009b; Dileepkumar et al. 2022; Prabagar et al. 2022), which can effectively satisfy the thermodynamic needs of different photo-redox processes (Wang et al. 2009a; Zhang et al. 2010). Nevertheless, the practical utilization of unmodified g-C_3_N_4_ is hindered by its low specific surface area, limited responsiveness to visible light (wavelengths less than 460 nm), and rapid recombination of photogenerated electron–hole pairs (Chen et al. 2023).

So, there are many efforts have been devoted to improve the g-C_3_N_4_ photocatalytic efficacy, including morphological controlled doping (Yu et al. 2015), metal deposition (Obregón et al. 2019), non-metal doping (Liu et al. 2010; Zhang and Antonietti 2010), construction of heterojunction or Z-scheme and S-scheme composites (She et al. 2017), introducing vacant defects (Niu et al. 2012) or designing porous structure (Li et al. 2014b). It has been demonstrated by Obregón et al. (2019); Wu et al. (2019); Yan et al. (2019) that the efficiency of photocatalytic materials can be enhanced by increasing the 2D surface area of g-C_3_N_4_, enlarging the number of active sites, and boosting mesoporosity in the photocatalysts. Furthermore, the photocatalytic efficiency of g-C_3_N_4_ can be enhanced by mixing with Quantum dots (QDs). Recently, Research has verified that merging g-C_3_N_4_ with QDs not only conquers the restrictions of each individual component but also synergistically combines their advantages (Chen et al. 2023).

Furthermore, the g-C_3_N_4_ 2D photocatalytic efficiency can be maximized via forming co-catalysts like Au or Ag or Pd metals on its surface. These metals can accumulate electrons on these surfaces after light irradiation at the Fermi level and then release them to degrade pollutants quickly (Chiu and Hsu 2017; Choi et al. 2017; Li et al. 2008). All these modifications leads to use g-C_3_N_4_ in different applications such as pollutants degradation (Yan et al. 2010), cleansing (Huang et al. 2014), water splitting (Wang et al. 2009b; Zhang et al. 2012), CO_2_ reduction (Huang et al. 2015; Wang et al. 2015) and sensitive detection (Jiang et al. 2016; Tian et al. 2013a, 2013b, 2013c). Besides, many efforts have been widely employed to enhance the photocatalytic performance of g-C_3_N_4_ 2D for potential applications, (including wastewater treatment, CO_2_ conversion, and PNP reduction) (El-Sheshtawy et al. 2019; Yang et al. 2013, 2017).

Herein, for the first time, we have combined 2D g-C_3_N_4_ with 0D g-C_3_N_4_ QDs and Ag as a cocatalyst to not only conquer the restrictions of each individual component but also synergistically combine their advantages. Thus, we have developed a new nanoporous brown photocatalyst (2% Ag/ g-C_3_N_4_ 2D/ g-C_3_N_4_ QDs) using a facile approach for completely fast reduction of PNP pollutants when exposed to visible light. The spectral, structural features, morphological properties and the photocatalytic efficiency of this nanocomposite were determined. The novel configuration of this photocatalyst demonstrated an extensive surface area characterized by a porous structure and a remarkable capability for absorbing a broad spectrum of visible light. The mechanisms of the charge separation/transfer and exceptional photocatalytic degradation of PNP were also proposed.

## Materials and methods

Dicyandiamide (DCDA), Pluronic (P123), and sodium citrate were purchased from Sigma-Aldrich, while silver nitrate (AgNO_3_) was supplied by Merck. Urea and Ethanol were obtained from Acros Organics-Fisher Scientific. Methanol was acquired from Chem-Lab Belgium. All materials were used without further purification.

### Manufacture of brown g-C_3_N_4_ mesoporous (g-C_3_N_4_ 2D)

The brownish g-C_3_N_4_ 2D mesoporous was synthesized by mixing 7 gm of DCDA with 0.9 gm of P123 in an alumina crucible. Then, the mixture was transferred inside a muffle oven following ramping procedures. The temperature increased to 250°C by 2 °C/min from room temperature for 2 h. After that, it was raised to 350 °C for 1 h with a temperature rate of 1.5 °C per min, and further, increased to 550 °C for 1 h with a temperature rate of 3 °C per min for about 4 h then it was cooled to ambient condition. The produced g-C_3_N_4_ 2D was calcinated at 450°C by 2 °C/min for 4 h.

### Fabrication of Ag/ g-C_3_N_4_ 2D by photodeposition method

Ag/g-C_3_N_4_ 2D was synthesized via a simple photodeposition process. Typically, various concentrations of Ag (0.5, 1, and 2 wt%) were added to g-C_3_N_4_ 2D (0.1 gm) dissolved in water/methanol (8:2 ml) and treated with visible light under stirring for 4 h. Then Ag/ g-C_3_N_4_ 2D was separated via centrifugation and washed by water. The Ag/ g-C_3_N_4_ 2D was used for further characterization after being dried at 80 ^o^C for 24 h.

### Synthesis of g-C_3_N_4_ QDs

G-C_3_N_4_ QDs can be prepared by this method (solid state method) without adding any solvent as follow: 1.68 mmol of urea (0.1015 gm) and 0.187 mmol of sodium citrate (0.055 gm) were blended using an agate mortar, in the presence of a few drops of ethanol to get a homogenous paste, followed by calcination at 80 °C. The produced powder was transferred into an autoclave and heated to 180°C over 1 h. The powder produced was dissolved in 5 ml water. The product was separated via centrifugation at 12000 rpm for 15 min. The supernatant was purified by dialyzing against ultra-pure for 24 h to obtain g-C_3_N_4_ QDs.

### Ternary brownish nanocomposite photocatalyst Ag/ g-C_3_N_4_ 2D / g-C_3_N_4_ QDs preparation

The ternary nanocomposite was synthesized by mixing 1ml of g-C_3_N_4_ QDs with 10 mg of Ag/ g-C_3_N_4_ 2D sample. The mixture was kept under stirring for 24 h. Then after drying at 60°C, the produced sample was denoted as Ag/ g-C_3_N_4_ 2D/ g-C_3_N_4_ QDs nanocomposite powder. For comparison, the nanocomposite without Ag was prepared by mixing 10 mg of g-C_3_N_4_ 2D with 1ml of g-C_3_N_4_ QDs to confirm the g-C_3_N_4_ 2D/ g-C_3_N_4_ QDs junction formation.

### Characterization

X-ray diffraction (XRD) was acquired using Rigaku Smart Lab X-ray diffractometer (40 kV/ 30 mA) with speed 0.5°/min using Cu K α radiation source with λ = 1.54 Å. Transmission electron microscope (TEM model. JEOL-JEM-2100F) was used to examine the morphological nanostructure. FE-SEM was employed to view topography details on the surface of the samples model: Quattro S FEG-SEM – Thermo Fisher, NL. Energy dispersive X-ray spectroscopy (EDX) was performed for elemental distribution.

The absorption and the diffuse reflection spectra (UV–Vis DRS) of the nanostructure powder were investigated by UV spectrophotometer (JASCO V-770) using BaSO_4_ as a standard sample, and the band gap was followed the Kubelka–Munk equation. The photoluminescence spectra were measured using JASCO FP-8600 spectrofluorometer.

The chemical surface structure of the samples was recorded using X-ray Photoelectron Spectroscopy (XPS, Thermo-Scientific Kα) technique with X-ray source–Al Kα micro-focused monochromator – varying spot size (30–400 m in 5 m steps), ion gun – energy range 100–4000 eV, vacuum system – 2 × 220 l/s turbo molecular pumps for entry and analysis chambers – auto-firing, filament TSP was used. Zeta potential measurements were determined by dynamic light scattering operated by Brookhaven, USA. The BET surface area analyzer (Nova 2000 series, Quantachrome Instruments, UK) was used for determining the powder's surface area, pore volume, and pore size distribution.

## Results and discussion

### Characterization of Ternary Ag/ g-C_3_N_4_ 2D / g-C_3_N_4_ QDs

**Scheme 1:**
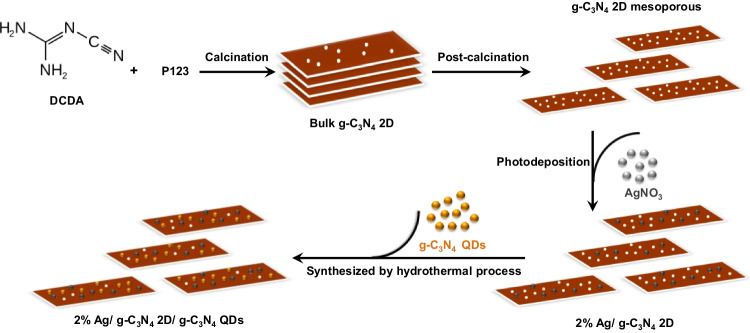
Synthesis of the brown 2% Ag/ g-C_3_N_4_ 2D/ g-C_3_N_4_ QDs nanocomposite

**Fig. 1:**
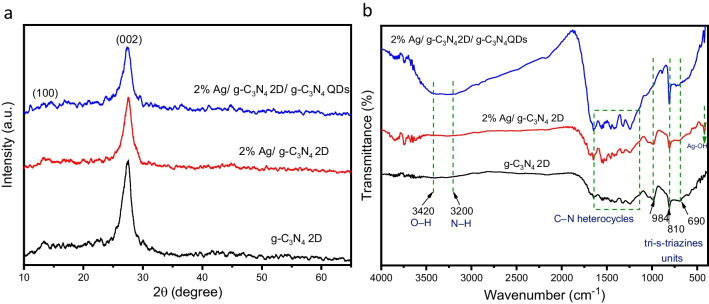
(**a**) XRD analysis of the as-prepared g-C_3_N_4_ 2D, 2% Ag/ g-C_3_N_4_ 2D and 2% Ag/ g-C_3_N_4_ 2D/ g-C_3_N_4_ QDs nanocomposites and (**b**) FT-IR spectra of g-C_3_N_4_ 2D, 2% Ag/ g-C_3_N_4_ 2D, 2% Ag/ g-C_3_N_4_ 2D/ g-C_3_N_4_ QDs

Scheme 1 illustrates the synthesis process of 2 wt% Ag/ g-C_3_N_4_ 2D/ g-C_3_N_4_ QDs nanocomposite via multiple stages. As indicated in Fig. 1 a, the XRD patterns of g-C_3_N_4_ display a strong diffraction peak at 2θ = 27.3° with a great intensity that can be attributed to the (002) interlayer stacking plane of aromatic conjugated C-N ring. The lower peak at 2θ = 13.3° is related to the (100) plane, which characteristics of the H-bonds responsible for preserving long-range atomic order in interlayers of tri-s-triazine rings (He et al. 2013; Xiu et al. 2014). The g-C_3_N_4_ peaks intensity decreases upon addition of 2% Ag, which reflects the distribution of Ag NPs on the surface of 2D g-C_3_N_4_. The intensity of these peaks is further reduced by adding g-C_3_N_4_ QDs to the surface of 2% Ag/ g-C_3_N_4_ 2D. The observed weak crystallinity of 2% Ag/ g-C_3_N_4_ 2D/ g-C_3_N_4_ QDs indicated that both Ag NPs and g-C_3_N_4_ QDs were well-dispersed into the g-C_3_N_4_ nanosheets. No diffraction peaks were observed for Ag NPs in the XRD pattern due to their small amount embedded in g-C_3_N_4_ 2D.

Figure 1 shows the FTIR spectra of the samples. g-C_3_N_4_ 2D exhibits fork-like peaks at 3200 cm^−1^ and 3420 cm^−1^ corresponding to the stretching vibrations of amine N–H and -NH_2_ groups, respectively (Vidyasagar et al. 2018). The observed peak at 1650 cm^−1^ may be attributed to the C-N stretching bonds, while the obtained peaks at 1543 cm^−1^, 1460 cm^−1^, 1328 cm^−1^ and 1248 cm^−1^ may be attributed to the heterocyclic C–N and C = N stretching vibrations (Xu et al. 2013a). Moreover, the presence of the tri-s-triazine rings in g-C_3_N_4_ is confirmed by the strong and sharp peak observed at 810 cm^−1^ (Li et al. 2014a; Zhang et al. 2013). However, the short peak placed at 1024 cm^−1^ is characterized by the C − N–C groups (Bojdys et al. 2008). After decorating the g-C_3_N_4_ surface with Ag NPs, the main specific peaks featured to g-C_3_N_4_ can be distinctively located in the 2wt% Ag/ g-C_3_N_4_ 2D composites, signifying that the overall g-C_3_N_4_ shape did not change, which agrees with XRD analysis. Furthermore, we observed that introducing Ag NPs into g-C_3_N_4_ 2D/ g-C_3_N_4_ QDs does not promote a change in the position of the characteristic peak of tri-s-triazine in g-C_3_N_4_ 2D (810 cm^−1^) suggesting that the configurational properties of the g-C_3_N_4_ 2D were not altered after the addition of Ag particles (Li et al. 2017). However, after adding 2wt% Ag NPs to g-C_3_N_4_ 2D a new peak appeared at 417 cm^−1^ due to (Ag–OH) which suggests the binding of Ag NPs to the mesoporous g-C_3_N_4_ 2D surface. More importantly, upon addition of g-C_3_N_4_ QDs to the 2% Ag/ g-C_3_N_4_ 2D sample, the N–H/-NH_2_ peak broadening at 3420 cm^−1^ directly relates to the hydroxyl (OH) groups involved in the 2% Ag/ g-C_3_N_4_ 2D/ g-C_3_N_4_QDs sample which originate from physically adsorbed water. The results indicate that Ag NPs were loaded on the surface of g-C_3_N_4_ 2D/ g-C_3_N_4_ QDs.

**Fig. 2:**
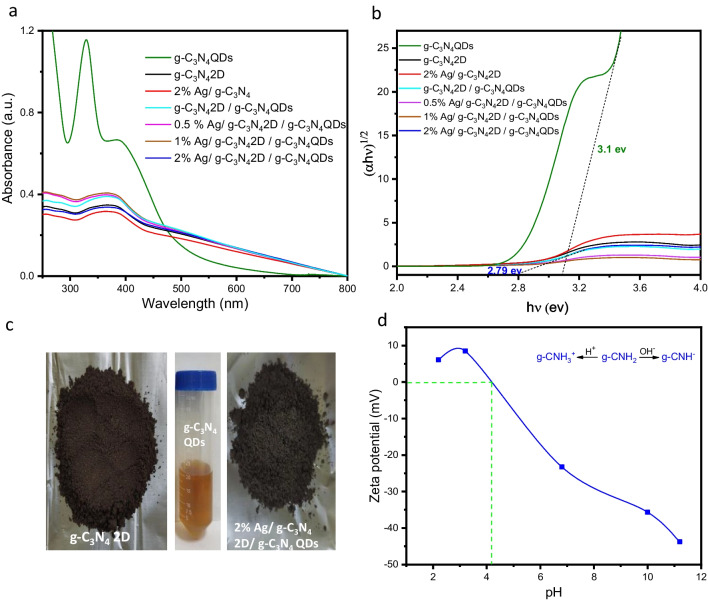
(**a**) UV–vis diffuse reflection spectra, (**b**) the band gap determination plots (**c**) show the photographs of each sample and (**d**) zeta potential as a function of pH values for 2% Ag/ g-C_3_N_4_ 2D/ g-C_3_N_4_ QDs

Figure 2 (a, b) shows the optical UV–Vis absorption spectra of the samples, it was observed that g-C_3_N_4_ 2D exhibited an absorption band at ~ 550 nm, g-C_3_N_4_ QDs exhibited broad absorption peaks at ~ 400 and ~ 550 nm. While, the onset absorption bands of Ag/ g-C_3_N_4_ 2D/ g-C_3_N_4_ QDs samples appeared at ~ 560 nm. On the other hand, the energy gap (E_g_) was determined using the Kubelka–Munk formula (Li et al. 2015). Figure 2 b, shows the calculated E_g_ values for g-C_3_N_4_ 2D, g-C_3_N_4_ QDs, 2% Ag/ g-C_3_N_4_, g-C_3_N_4_ QDs/ g-C_3_N_4_ 2D, 0.5% Ag/ g-C_3_N_4_ 2D/ g-C_3_N_4_ QDs, 1% Ag/ g-C_3_N_4_ 2D/ g-C_3_N_4_ QDs and 2% Ag/ g-C_3_N_4_ 2D/ g-C_3_N_4_ QDs were 2.82, 3.10, 2.80, 2.70, 2.71, 2.73 and 2.79 eV, respectively. All samples showed a brown color as shown in Fig. 2 c. The small change in the band gaps after the addition of Ag NPs confirms their embedding on the surface of g-C_3_N_4_ 2D/ g-C_3_N_4_ QDs.

The surface charge and the stability of the 2% Ag/g-C_3_N_4_ 2D /g-C_3_N_4_ QDs suspension were clarified by measuring their ζ potential at different pH values. As displayed in Fig. 2 d, the ζ potential of the 2% Ag/ g-C_3_N_4_ 2D / g-C_3_N_4_ QDs at pH values; 2.2, 3.2, 6.8, 10.4 and 11.2 were 6.11 mV, 8.4 mV, -22.5 mV, -35.6 mV and -42.6 mV, respectively. The results reveal that the ζ potential value of 2% Ag/ g-C_3_N_4_ 2D / g-C_3_N_4_ QDs increases gradually with rising pH and its isoelectric point (IEP = pH at ζ potential equal zero) is 4.2 (Torad et al. 2021; Zhu et al. 2015). The ζ potential outcomes also revealed that 2% Ag/g-C_3_N_4_ 2D/ g-C_3_N_4_ QDs possess high negative charges above the IEP and have positive charges at pH values lower than this point. The surfaces with greatly negative charges provide a high electrostatic repulsion between the nanosheets resulting in better stability and homogeneity in aqueous solutions as recently (Milaneze et al. 2016) it was reported that the NPs exhibit ζ potential values (> + 25) mV or (< − 25 mV) display enough electrostatic repulsion to keep their solution stability. Hence, this can be highly beneficial for studying their optical and sensing characteristics. Thus, the examined nanocomposite provided high stability in the alkaline media.

**Fig. 3:**
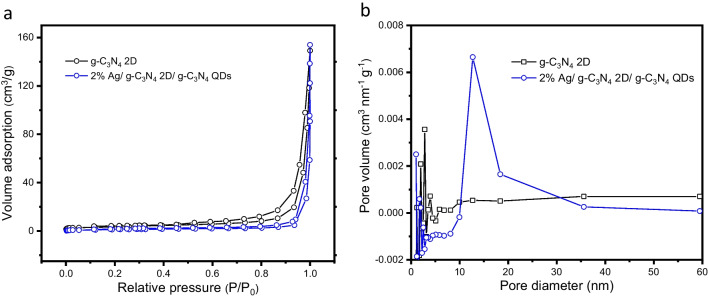
(**a**) N_2_ absorption–desorption isotherms and (**b**) the pore size distribution curves of g-C_3_N_4_ 2D and 2% Ag/ g-C_3_N_4_ 2D/ g-C_3_N_4_ QDs

The specific surface area, pore volume and size distribution were studied for g-C_3_N_4_ 2D and 2% Ag/ g-C_3_N_4_ 2D/ g-C_3_N_4_ QDs samples using the N_2_ adsorption–desorption isotherm. As shown in Fig. 3 a, the samples exhibited type IV isotherm that is accompanied by H_3_ hysteresis loops, suggesting their presence as the predominant mesopores structure of the obtained nanocomposite. Due to the significant amount of (CO_2_ and NH_3_) gases emitted from the precursor materials during calcination, mesoporosity has developed in the samples under investigation. The adsorbent amount of N_2_ molecules on the surface of 2% Ag/ g-C_3_N_4_ 2D/ g-C_3_N_4_ QDs has been decreased as compared to g-C_3_N_4_ 2D as displayed in Fig. 3 a. This is due to some Ag NPs having been adsorbed in the surface of g-C_3_N_4_ 2D. So, the surface area of the 2% Ag/ g-C_3_N_4_ 2D/ g-C_3_N_4_ QDs has been decreased from 150 m^2^g^−1^ to 140 m^2^g^−1^ upon the addition of Ag NPs and g-C_3_N_4_ QDs. As shown in Fig. 3 b, the average diameter of g-C_3_N_4_ 2D pore size was determined from the distribution curves using the BJH model to be about 4 nm. This highly porous structure of g-C_3_N_4_ 2D comes from the introducing holding sequences at intermediate temperatures around the surfactant decomposition (P123) during the calcination step to avoid volatilization (Wang et al. 2010). However, we observed broad and higher porosity for 2%Ag/ g-C_3_N_4_ 2D/ g-C_3_N_4_ QDs with pore size distribution of ~ 13 nm suggesting that heterojunction occurred between the g-C_3_N_4_ 2D and g-C_3_N_4_ QDs.

**Fig. 4:**
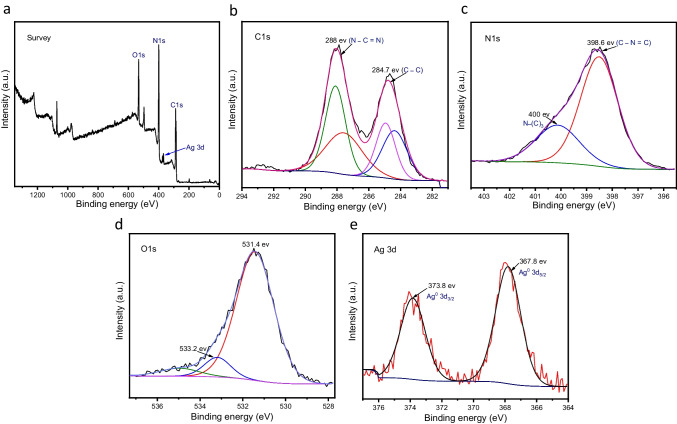
The XPS spectra of the 2% Ag/ g-C_3_N_4_ 2D/ g-C_3_N_4_ QDs sample for the (**a**) survey spectrum, (**b**) C1s, (**c**) N1s, (**d**) O1s and (**e**) Ag 3d region

To elucidate the chemical oxidation state and the structure of 2% Ag/ g-C_3_N_4_ 2D/ g-C_3_N_4_ QDs sample, we performed XPS measurements. Figure 4 a gives the distribution of Ag, N, C, and O species in the nanocomposite. As seen in Fig. 4 b, the observed two peaks in the C 1 s XPS spectra at 284.7 eV and 288 eV are characterized to sp^2^ C–C bonds and sp^2^ N–C = N bonds of the graphitic domains and the s-triazine units, respectively (Xu et al. 2013b). The observed peaks in the N 1 s spectrum at 398.6 eV and 400 eV is corresponding to sp^2^ (C–N = C) in the s-triazine unit and the sp^3^—located in N–(C)_3_, respectively as shown in Fig. 4 c (Xu et al. 2013c; Zhang et al. 2013). Furthermore, as shown in Fig. 4 d, we observed low amount of oxygen species probably due to two prominent peaks (in the O 1 s spectra) within 531.4 eV and 533.2 eV to chemisorbed oxygen species on the surface (Veerakumar et al. 2018). Moreover, Fig. 3 e showed the characteristic peaks for Ag 3d species. It displayed formation of two characteristic peaks at 367.8 eV and 373.8 eV, belonging to the metallic (Ag^0^) 3d _3/2_ and (Ag^0^) 3d_5/2_, respectively, which is consistent with the literature value of Ag NPs (An et al. 2012).

**Fig. 5:**
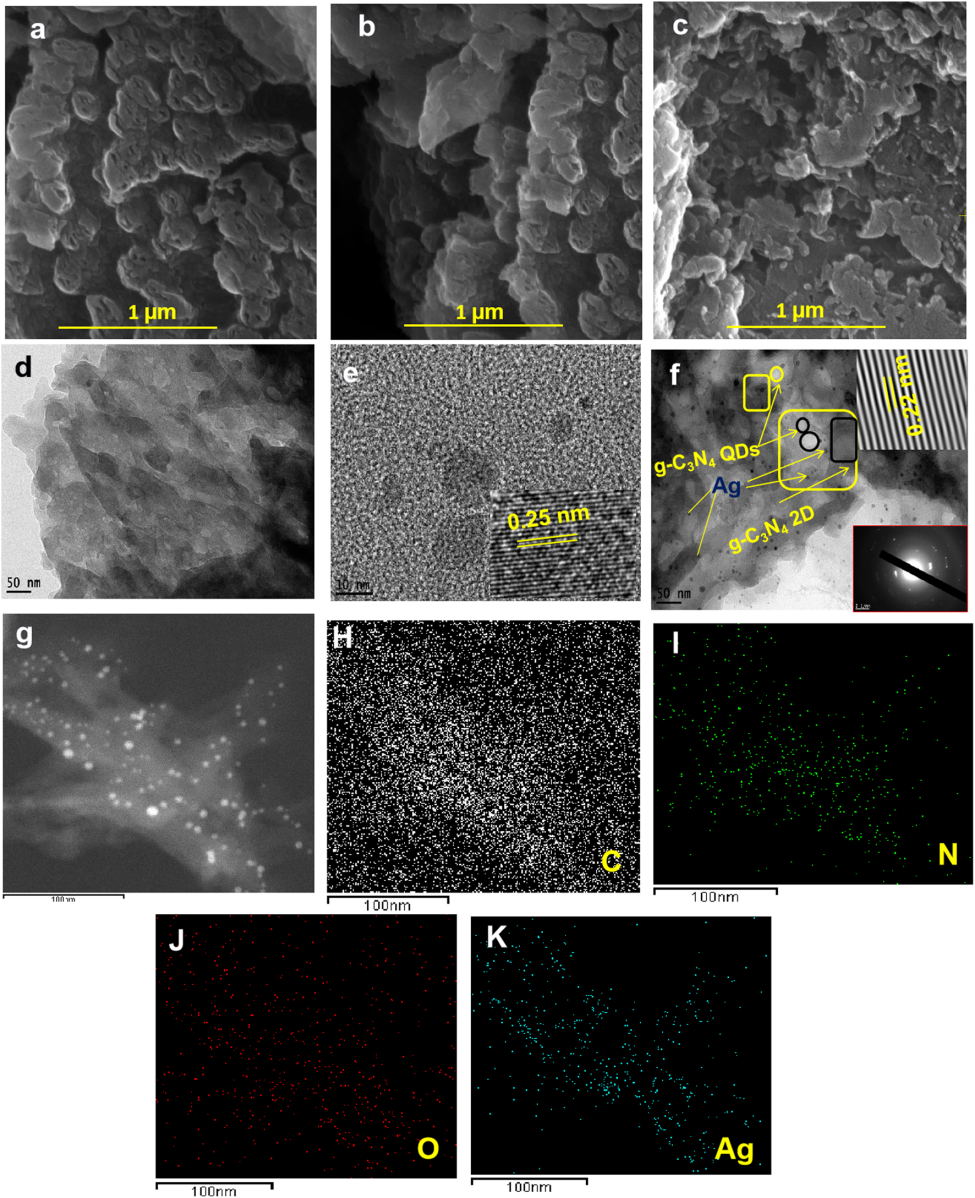
FE-SEM images of (**a**) g-C_3_N_4_ 2D, (b) 2% Ag/ g-C_3_N_4_ 2D and (**c**) 2% Ag/ g-C_3_N_4_ 2D/ g-C_3_N_4_ QDs samples, HRTEM images (**d**) g-C_3_N_4_ 2D, (e) g-C_3_N_4_ QDs and (**f**) 2% Ag/ g-C_3_N_4_2D/ g-C_3_N_4_QDs samples and (**g**) high angle annular dark field-scanning transmission electron microscopy (HAADF-STEM) image, and corresponding elemental mapping images of 2% Ag/ g-C_3_N_4_ 2D/ g-C_3_N_4_ QDs sample (H–K)

The surface morphology, structural analysis, and size distribution of the g-C_3_N_4_ 2D, g-C_3_N_4_ QDs, and 2%Ag/ g-C_3_N_4_ 2D/ g-C_3_N_4_ QDs were performed by FE-SEM and HR-TEM analysis as shown in Fig. 5 (a-k). FE-SEM images of g-C_3_N_4_ 2D show distributed multiple lamellar structures with numerous irregular worm-like holes; reflecting the pore structures within these nanosheets as revealed in Fig. 5 a. In comparison to the naked g-C_3_N_4_ 2D, the 2% Ag/ g-C_3_N_4_ 2D and heterojunction ternary 2% Ag/ g-C_3_N_4_ 2D/ g-C_3_N_4_ QDs mesoporous nanocatalyst, exhibited a decrease in the porosity as shown in Fig. 5 (b, c). These results revealed that the Ag NPs are loaded on the surface and/or in between the g-C_3_N_4_ 2D/ g-C_3_N_4_ QDs heterojunction.

Furthermore, the HR-TEM images of g-C_3_N_4_ 2D also showed laminar multilayer morphology characteristic for the graphitic nature, consistent with the earlier observation (Kang et al. 2016), and the observed porous nanosheets are thinner with lateral thickness below 50 nm as displayed in Fig. 5 d (Veerakumar et al. 2018; Vidyasagar et al. 2018). The HR-TEM images of the as-synthesized g-C_3_N_4_ QDs monodisperse (Fig. 5 e)), showed the appearance of distinct clusters that were individually distributed without aggregation and exhibit sizes ranging from 5 to 15 nm, and its SAED image (inset Fig. 5 e) showed the crystal interplanar distance of 0.25 nm**.** Contrariwise, the images of HR-TEM (Fig. 5 f) of the 2% Ag/ g-C_3_N_4_ 2D/ g-C_3_N_4_ QDs show Ag NPs well-tightly distributed around the g-C_3_N_4_ matrix's surface. The observed average size of Ag NPs is in the range 10–15 nm, suggesting a successful fabrication of Ag NPs on sheet matrix. The inset of Fig. 5 f shows the crystalline structure of Ag NPs within (d = 0.22 nm) and the SEAD displays a concentric rings because of the g-C_3_N_4_ QDs and g-C_3_N_4_ 2D heterojunction (Li et al. 2017; Yu et al. 2020). The results suggest that Ag NPs are successfully adsorbed on the surface of g-C_3_N_4_ 2D/ g-C_3_N_4_ QDs.

Figure 5 (g-k) shows the characteristics elemental mappings and distribution of 2% Ag/ g-C_3_N_4_ 2D/ g-C_3_N_4_ QDs, using EDX-TEM scanning that shows the content of C (26.54%), N (61%0.76), O (10.22%), and Ag (1.48) mass %. Analysis results showed that Ag particles are successfully deposited on the g-C_3_N_4_ 2D matrix which reflects their dense and uniform distribution throughout the whole composite surface.

### Catalytic and photocatalytic ability of 2%Ag/ g-C_3_N_4_ 2D/ g-C_3_N_4_ QDs nanocatalyst

**Fig. 6:**
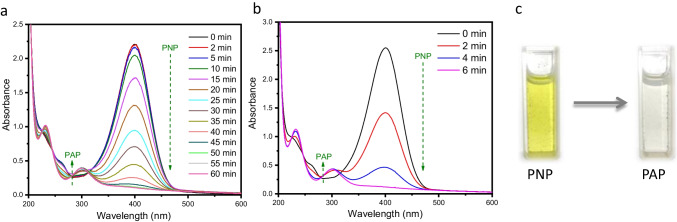
Time dependent UV–vis absorption spectra of (**a**) catalytic reaction of PNP to PAP over 2% Ag/ g-C_3_N_4_ 2D/ g-C_3_N_4_ QDs and (**b**) under direct visible light. (**c**) The conversion of the PNP yellow color to the PAP colorless

The hydrogenation reaction of PNP in the presence of a reducing agent is used as a representative reaction to study both the catalytic and photocatalytic reduction action of the g-C_3_N_4_ 2D, g-C_3_N_4_ QDs, and the three different ternary nanocomposites of Ag/ g-C_3_N_4_ 2D/ g-C_3_N_4_ QDs nanocatalyst. Figure 6 shows the UV–Vis absorption spectra of PNP upon addition of a reducing agent (NaBH_4_).

There is a spectral shift on the absorption peak from 317 to 400 nm consistent with the formation of nitrophenolate ion. As shown in Fig. 6, we have studied the effect of adding ternary photocatalyst to PNP, and results showed a decrease in the absorption intensity of the 400 nm absorption peak which completely disappeared after 60 min and a novel peak appeared at 300 nm due to the produced p-aminophenol (PAP). In this system, we observed a change in the solution color from yellowish to colorless (Fig. 6 c).

The kinetic behavior of the reduction of PNP to PAP follows the empirical pseudo/first order kinetic equation: ln ([C]**/**[C]_o_) = kt, where C_0_ and C are the concentrations of PNP at zero and t time, respectively. The plotting of Ln(C/C_0_) versus time is linear and the rate constant (k) can be obtained from the slope.

**Fig. 7:**
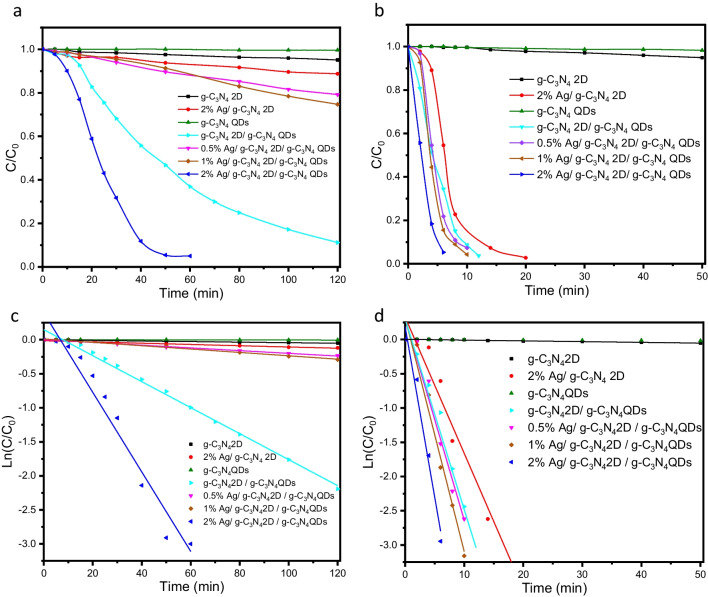
Plots of C/C_0_ versus reaction time for the catalytic reduction of NP to AP over different catalysts (**a**) in the dark, (**b**) under direct visible light, and ln(C/C_0_) versus reaction time for (**c**) in the dark and (**d**) under direct visible light

As demonstrated in Fig. 7 (a-d) and Table 1, it is evident that both g-C_3_N_4_ 2D and g-C_3_N_4_ QDs display a slow kinetic rate and then poor catalytic activity, whereas after photodeposition of Ag NPs into the g-C_3_N_4_ 2D surface, the Ag NPs initiate the reduction process and there is a significant increase in the reaction rate with increasing the content of Ag NPs in the ternary catalyst followed by the degradation of PNP. The complete decomposition takes 60 min in the dark after the adsorption of 2% Ag NPs on the g-C_3_N_4_ 2D /g-C_3_N_4_ QDs surface. After exposure to the PNP solution which contains the catalyst for solar light, the degradation of the PNP to the PAP is dramatically enhanced. The reduction rate is greatly accelerated by exposing visible light on (2% Ag/ g-C_3_N_4_ 2D /g-C_3_N_4_ QDs)/ PNP/ NaBH_4_ structure.

**Table 1:** Reaction rate of PNP reduction using different catalysts in dark and under visible light

Catalyst	PNP → PAP k/ min^−1^	
	Absence of visible light	Presence of visible light
g-C_3_N_4_ 2D	0.00041	0.00111
2% Ag/ g-C_3_N_4_ 2D	0.00092	0.19888
g-C_3_N_4_ QDs	0.00004	0.00036
g-C_3_N_4_ 2D/ g-C_3_N_4_ QDs	0.0191	0.27838
0.5% Ag/ g-C_3_N_4_ 2D / g-C_3_N_4_ QDs	0.00201	0.29383
1% Ag/ g-C_3_N_4_ 2D / g-C_3_N_4_ QDs	0.00248	0.34123
2% Ag/ g-C_3_N_4_ 2D / g-C_3_N_4_ QDs	0.05844	0.49729

As shown in Fig. 6 and Table 1, the reduction of PAP with 2%Ag/ g-C_3_N_4_ 2D /g-C_3_N_4_ QDs photocatalyst under visible light only takes 6 min, whereas doing it in darkness with the same catalyst takes 60 min**.** As a result, the catalytic effect of light accelerates the reduction rate constant by 10 times that in the dark. As shown in Table 1, these data suggest that the designed photocatalyst enhances the conversion of PNP to the corresponding PAP compared to that of previously reported work **(**Table 2**)** (Jiang et al. 2021; Qu et al. 2022).

**Table 2:** illustrates the previous studies data on the different times of PNP reduction with different photocatalysts compared to the current work

Photocatalyst	Time (min)	Decomposition (%)	Ref
5%Ag/ g-C_3_N_4_	120	98	(Yang et al. 2013)
4%TiO_2_/ CN	100	95.8	(Qu et al. 2022)
20%Ag/ g-C_3_N_4_/ CNF	50	100	(Yu et al. 2017)
NiCo_2_O_4_/ g-C_3_N_4_	30	91.4	(Jiang et al. 2021)
1.5 Ag/ g-C_3_N_4_/ V_2_O_5_	8	100	(El-Sheshtawy et al. 2019)
2% Ag/ g-C_3_N_4_ 2D / g-C_3_N_4_ QDs	6	100	(This work)

### Mechanism for catalytic reaction

The catalytic performance of 2%Ag/ g-C_3_N_4_ 2D/ g-C_3_N_4_ QDs nanomaterial is increasing the degradation of PNP both in dark and during exposure to light. In the case of dark, the electron transfer mechanism is responsible for the catalytic reduction (Zhao et al. 2015). Initially, PNP and the reducing agent (NaBH_4_) were both adsorbed on the porous g-C_3_N_4_ nanocatalyst. This is followed by the oxidation of NaBH_4_, which leads to the production of borate ions (BH_4_^−^) and the release of free electrons essential for PNP breakdown (Zhao et al. 2015).

**Fig. 8:**
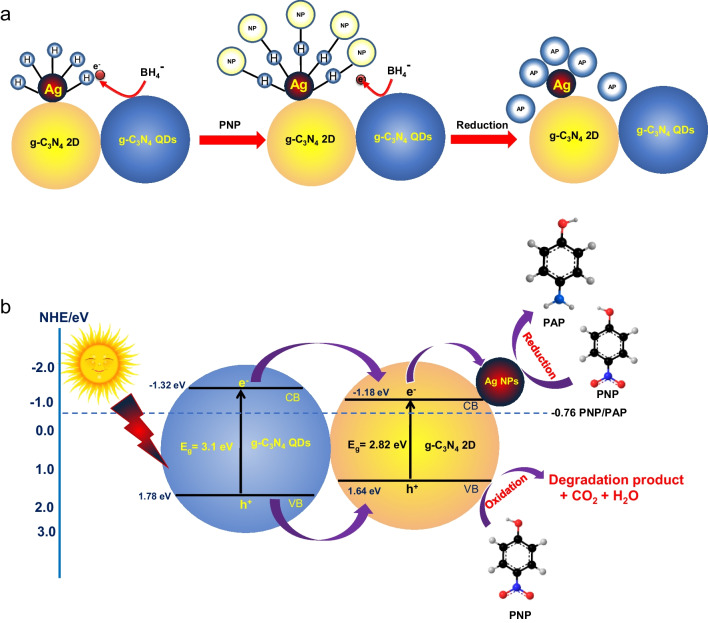
Proposed mechanism for the effect of 2%AgNPs loaded on g-C_3_N_4_ 2D/ g-C_3_N_4_ QDs heterojunction in the catalytic/photocatalytic conversion of p-nitrophenol to p-aminophenol (**a**) in the dark and (**b**) under visible light

Due to the formation of active hydrogen atoms on the surface of the Ag NPs, which react with the PNP and transform it into PAP as shown in Fig. 8 a, the degradation rate was significantly enhanced after addition of Ag NPs on the surface of g-C_3_N_4_ 2D followed by addition of g-C_3_N_4_ QDs**.**

### Mechanism for photocatalytic reduction of PNP

The reduction of PNP to PAP using 2% Ag/ g-C_3_N_4_ 2D/ g-C_3_N_4_ QDs as photocatalyst proceeds via a charge separation mechanism (Hong et al. 2016). The CB and VB potentials were determined using the following equations (Zhang et al. 2015):

1 $${\mathrm{E}}_{\mathrm{CB}}=\upchi -{\mathrm{E}}^{\mathrm{e}}-0.5{\mathrm{E}}_{\mathrm{g}}$$

2 $${\mathrm{E}}_{\mathrm{VB}}={\mathrm{E}}_{\mathrm{CB}}+{\mathrm{E}}_{\mathrm{g}}$$

While E_CB_, E^e^, and E_VB_ represent the conduction band potential, released electrons energy compared to standard hydrogen potential (4.5 eV) and the valence band potential (Morrison and Morrison 1980) respectively, the electronegativity of semiconductors is χ can be determined using (Yuan et al. 2014):

3 $$\upchi ={\left[\upchi {\left(\mathrm{A}\right)}^{\mathrm{a}}\upchi {\left(\mathrm{B}\right)}^{\mathrm{b}}\upchi {\left(\mathrm{C}\right)}^{\mathrm{c}}\right]}^{1/\left(\mathrm{a}+\mathrm{b}+\mathrm{c}\right)}$$

where a, b and c represent the number of individual atoms.

The determined conduction band and valence band for g-C_3_N_4_ 2D were -1.18 eV and 1.64 eV, respectively, and for the g-C_3_N_4_ QDs were -1.32 eV and 1.78 eV, respectively. Electron/hole species formed when visible light is used to irradiate a 2% Ag/g-C_3_N_4_ 2D/g-C_3_N_4_ QDs sample. These photo-produced electrons in the g-C_3_N_4_ QDs CB levels shifted to g-C_3_N_4_ 2D CB levels since the g-C_3_N_4_ QDs CB levels are higher than g-C_3_N_4_ 2D CB levels in keeping with the Type I mechanism of electron/hole separation as shown in Fig. 8 b. Moreover, the photogenerated holes inside the g-C_3_N_4_ QDs valence band are shifted to the g-C_3_N_4_ 2D due to the higher potential energy of the g-C_3_N_4_ QDs VB levels. As a result, the g-C_3_N_4_ 2D conduction band drives the reduction process, and its VB regulates the oxidation process. When the CB of the photocatalyst is sufficiently negative (E = -1.18 eV compared to hydrogen electrode) to reduce the PNP to the corresponding PAP (E =—0.76 eV). Moreover, the g-C_3_N_4_ 2D accumulated electrons in the CB are moving on the Ag NPs surface which performs as electron storage and is responsible for the complete PNP reduction to PAP as shown in Fig. 8 b. As well as the g-C_3_N_4_ 2D holes within the valence band are responsible for some species of PNP oxidizing CO_2_ (2 µmole, measured using GC) as presented in Fig. 8 b. To confirm the importance of g-C_3_N_4_ QDs in this photocatalytic system, only 2% Ag/g-C_3_N_4_2D is used as a photocatalyst for the reduction of PNP to PAP. Surprisingly, the results showed that the PNP was completely reduced to PAP within 20 min. Thus, we can conclude that the addition of g-C_3_N_4_ QDs (a photosensitizer) to the 2% Ag/ g-C_3_N_4_ 2D system enhances the reduction of PNP to PAP within 6 min as shown in Fig. 7 b. Likewise, different amounts of Ag NPs were loaded into the g-C_3_N_4_ 2D to prove the importance of Ag NPs. As can be seen from Fig. 7 (b, d) and Table 1, the observed data showed that increasing the amount of Ag NPs from 0.5% to 2% increases the photocatalytic reduction of PNP to PAP. Thus, we concluded that 2% Ag/ g-C_3_N_4_ 2D/ g-C_3_N_4_ QDs photocatalyst is the optimum sample for enriching the PNP reduction ability of the corresponding PAP in a relatively short time (6 min).

**Fig. 9:**
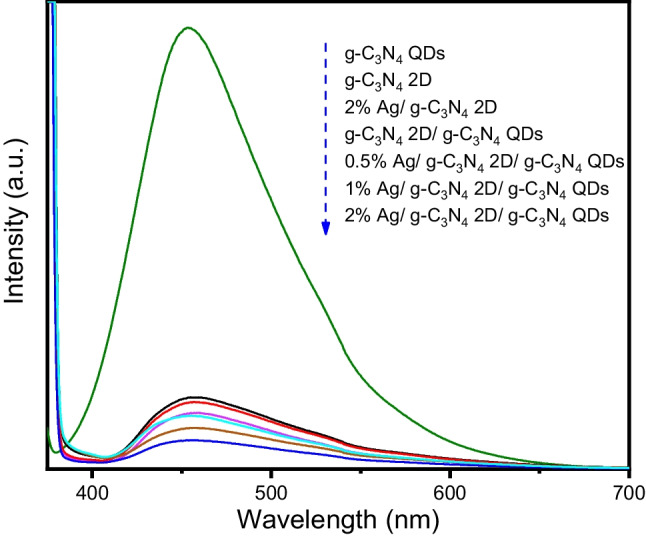
The photoluminescence spectra of g-C_3_N_4_ QDs, g-C_3_N_4_ 2D, 2% Ag/ g-C_3_N_4_ 2D, g-C_3_N_4_ 2D/ g-C_3_N_4_ QDs, 0.5% Ag/ g-C_3_N_4_ 2D/ g-C_3_N_4_ QDs, 1% Ag/ g-C_3_N_4_ 2D/ g-C_3_N_4_ QDs and 2% Ag/ g-C_3_N_4_ 2D/ g-C_3_N_4_ QDs samples

To address the role of 2% Ag/ g-C_3_N_4_ 2D/ g-C_3_N_4_ QDs in accelerating the photocatalytic ability for complete reduction of PNP compared to other samples. Photoluminescence (PL) study is performed as displayed in Fig. 9. The PL analysis helps determine in inspecting the efficiency of separation activity of charge carriers within this photocatalyst and investigating the electron/hole pair lifetime. All fabricated nanocomposites display a peak at 453 nm, indicating the trap energy state-defect emission. Furthermore, according to the obtained data, the most intense emitted portion is observed for g-C_3_N_4_ QDs sample and its intensity decreased after creating a heterojunction with g-C_3_N_4_ 2D as well as reduced by adding more Ag NPs quantity which considers the main active sites in g-C_3_N_4_ 2D/ g-C_3_N_4_ QDs nanocatalyst. This illustrates the importance of Ag NPs in reducing the recombination rate of the electron/hole pair in the 2% Ag/ g-C_3_N_4_ 2D/ g-C_3_N_4_ QDs photocatalyst, resulting in superior photocatalytic efficiency as previously mentioned. The PL data were determined to give more proof of the photocatalytic efficacy improvement of the fabricated photocatalysts.

On the other hand, to confirm the durability and stability of the produced 2% Ag/ g-C_3_N_4_ 2D/ g-C_3_N_4_ QDs nanocomposite in practical applications, we investigated its recyclability through four successive cycles of 4-NP photoreduction under illumination (refer to Fig. S1). Following each run, the solid catalyst was separated from the mixture via centrifugation, subsequently dried for employment in the subsequent cycle. The outcome illustrated a marginal reduction in the photocatalytic reduction efficacy, decreasing from 100 to 98% after four times. Furthermore, XRD analysis was executed on the reutilized sample, as depicted in Fig. S2. The XRD findings demonstrated an unaltered XRD pattern, affirming the impeccable reusability and stability of the synthesized 2% Ag/ g-C_3_N_4_ 2D/ g-C_3_N_4_ QDs nanocomposite. Additionally, the FTIR examination (Fig. S3), for the same sample revealed typical spectra before and after usage of the % Ag/ g-C_3_N_4_ 2D/ g-C_3_N_4_ QDs nanocomposite, indicating its stability.

## Conclusion

In summary, this research presents novel nano-heterostructure brown g-C_3_N_4_ based materials for enhancing the photocatalytic reactivity, while ternary mesoporous Ag/ g-C_3_N_4_ 2D/ g-C_3_N_4_ QDs photocatalysts are easily synthesized with different Ag NPs percentages via the photodeposition method. The characterization of the prepared photocatalyst components by various morphological and spectroscopic techniques proved that the Ag/ g-C_3_N_4_ 2D/ g-C_3_N_4_ QDs photocatalysts have a mesoporous property with a wide absorption range in the visible light region. As well as, it evidenced that Ag NPs well distrusted on the surface g-C_3_N_4_ matrix. Moreover, the data illustrate and prove the considerable photocatalytic improvement of the ternary nanocomposite (2% Ag/ g-C_3_N_4_ 2D/ g-C_3_N_4_ QDs) via electron/hole pairs separation. The fabricated nanocatalysts were tested to evaluate their ability to remove PNP with or without visible light. The ternary photocatalysts show significantly enhanced photocatalytic properties for the reduction of PNP in a short period of time (6 min) when subjected to visible light. According to the recorded findings, the fabricated ternary nanocomposite could be one of the potential solar light-induced nano-heterostructure photocatalysts for different environmental applications.

### Supplementary Information

Below is the link to the electronic supplementary material.

Supplementary file1 (DOCX 315 KB)

## Data Availability

All data are available from the corresponding author on request.
