# Supplementary material for: Efficient photocatalytic reduction of p-nitrophenol under visible light irradiation based on Ag NPs loaded brown 2D g-C3N4 / g-C3N4 QDs nanocomposite
Source: Environ Sci Pollut Res Int. 2023 Oct 24;30(55):117909–22. doi: 10.1007/s11356-023-30010-z (PMC10682077; doi:10.1007/s11356-023-30010-z)
Supplement: Supplementary file 1 — Supplementary file1 (DOCX 315 KB) [file 11356_2023_30010_MOESM1_ESM.docx]

**Efficient photocatalytic** **reduction of p-nitrophenol under visible light irradiation based on Ag NPs loaded brown 2D g-C_3_N_4_ / g-C_3_N_4_ QDs nanocomposite**

Sandy Maged, Ola M. El‑Borady, Hamza El-Hosainy, Maged El-Kemary*

Nano sensor Group, Institute of Nanoscience and Nanotechnology, Kafrelsheikh University, 33516, Kafr ElSheikh, Egypt

Supporting information:

Fig. S2. XRD spectra for 2% Ag/ g-C_3_N_4_ 2D/ g-C_3_N_4_ QDs nanocomposite before and after reuse.

Fig. S3. The FTIR spectra for 2% Ag/ g-C_3_N_4_ 2D/ g-C_3_N_4_ QDs nanocomposite before and after reuse.
